# Diagnostic accuracy of the STRATIFY clinical prediction rule for falls: A systematic review and meta-analysis

**DOI:** 10.1186/1471-2296-13-76

**Published:** 2012-08-07

**Authors:** Jennifer Billington, Tom Fahey, Rose Galvin

**Affiliations:** 1 HRB Centre for Primary Care Research, Department of General Practice, Royal College of Surgeons in Ireland, 123 St. Stephens Green, Dublin 2, Republic of Ireland

**Keywords:** Falls assessment, STRATIFY, Sensitivity and specificity, Systematic review, Meta-analysis

## Abstract

**Background:**

The STRATIFY score is a clinical prediction rule (CPR) derived to assist clinicians to identify patients at risk of falling. The purpose of this systematic review and meta-analysis is to determine the overall diagnostic accuracy of the STRATIFY rule across a variety of clinical settings.

**Methods:**

A literature search was performed to identify all studies that validated the STRATIFY rule. The methodological quality of the studies was assessed using the Quality Assessment of Diagnostic Accuracy Studies tool. A STRATIFY score of ≥2 points was used to identify individuals at higher risk of falling. All included studies were combined using a bivariate random effects model to generate pooled sensitivity and specificity of STRATIFY at ≥2 points. Heterogeneity was assessed using the variance of logit transformed sensitivity and specificity.

**Results:**

Seventeen studies were included in our meta-analysis, incorporating 11,378 patients. At a score ≥2 points, the STRATIFY rule is more useful at ruling out falls in those classified as low risk, with a greater pooled sensitivity estimate (0.67, 95% CI 0.52–0.80) than specificity (0.57, 95% CI 0.45 – 0.69). The sensitivity analysis which examined the performance of the rule in different settings and subgroups also showed broadly comparable results, indicating that the STRATIFY rule performs in a similar manner across a variety of different ‘at risk’ patient groups in different clinical settings.

**Conclusion:**

This systematic review shows that the diagnostic accuracy of the STRATIFY rule is limited and should not be used in isolation for identifying individuals at high risk of falls in clinical practice.

## Background

Falls are a significant cause of morbidity and mortality and frequently lead to lasting loss of mobility, fractures and limitations in social participation [1-3]. The risk of falling increases with age and the prevalence of falls varies in different clinical settings [2]. In the Irish context, the inpatient cost of fall-related hospitalisations among older people is currently estimated at €59 million and falls among inpatients accounts for 32% of incident reports in UK hospitals [4,5]. Commonly identified risk factors for falls in hospitalised patients include gait instability, altered mental state, urge incontinence, a past history of falling, use of certain medications (particularly sedatives and hypnotics), use of restraints and environmental factors [6,7].

A number of clinical prediction rules (CPRs) have been derived to assist clinicians in identifying patients at risk of falling. The STRATIFY clinical prediction rule (St. Thomas Risk Assessment Tool in Falling elderly inpatients) displayed in Additional file 1: Table S1, consists of five items that address risk factors for falling including past history of falling, patient agitation, visual impairment affecting everyday function, need for frequent toileting, and transfer ability and mobility [8]. The STRATIFY rule yields a possible score between 0 and 5 (each item scoring 1 if present or 0 if absent). The transfer and mobility item on the STRATIFY rule combines the transfer and mobility sections of the Barthel Index and a score of 3 or 4 on the transfer and mobility sections of the Barthel Index is associated with a higher fall risk than a lower or higher score, thus scoring 1 point on the STRATIFY rule. The CPR was originally derived using a case control study design in mixed acute/rehabilitation geriatric wards of a UK urban teaching hospital. A score of ≥2 indicates a high risk of falls. The STRATIFY CPR is commonly used as a falls risk assessment tool in clinical practice and since the publication of the derivation study in 1997, several studies have validated the STRATIFY rule across a variety of clinical settings. A previous systematic review and meta-analysis demonstrated that the predictive accuracy of the STRATIFY rule in a geriatric setting was limited with overall sensitivity and specificity estimates of 0.67 (95% CI 0.61–0.74) and 0.51 (95% CI 0.43 – 0.59) respectively [5]. However, only data from four studies were included in the meta-analysis and a number of further validation studies have been completed in the interim. We conducted a systematic review and meta-analysis to determine the totality of evidence in relation to the overall diagnostic accuracy of the STRATIFY rule across a variety clinical settings.

## Methods

### Search strategy

The PRISMA guidelines for reporting of systematic reviews and meta-analysis were followed to conduct this review. The Cochrane handbook for diagnostic test accuracy studies was also referenced. We aimed to identify all studies that validated the STRATIFY rule irrespective of setting, language or study design. An online literature search was conducted in July 2011 and included the following search engines: Pubmed, EMBASE, EBSCO, Science Direct, CINAHL and Cochrane library. The databases were searched using a combination of the following keywords and MeSH terms: ‘STRATIFY’, ‘falls’, ‘risk assessment’ and ‘clinical assessment tool’. The search was supplemented by hand searching references of retrieved articles and searching Google Scholar. The original STRATIFY derivation paper was published in 1997 [8], therefore studies published from 1997 – July 2011 were included in our analysis.

### Study selection and data extraction

Studies were included if they met the following inclusion criteria: 1) Prospective or retrospective cohort studies; 2) Studies that validated the STRATIFY CPR; 3) Studies that included hospital inpatients, rehabilitation patients and nursing home inpatients; 4) Studies that recorded a subsequent fall. We used the following definition of a fall: an unexpected event in which the patient comes to rest on the ground, floor or lower level. Two reviewers (JB, RG) read the titles and/or abstracts of the identified references and eliminated irrelevant studies. Studies that were considered eligible for inclusion were read fully in duplicate and their suitability for inclusion was independently determined by both RG and JB. Disagreements were managed by consensus.

Data was extracted on study setting, patient demographics (age, gender), population type (e.g. geriatric rehabilitation patients, stroke patients), length of follow up, details of the person administering the STRATIFY rule, total number of episodes of falls (falls) and the number of individuals who fell (fallers). For the purposes of this paper, the unit of analysis was the patient or ‘faller’ rather than each ‘fall’ to avoid duplication bias. This is consistent with the main purpose of the STRATIFY CPR, to identify individuals who are at high risk of falling. Authors were contacted to provide further information on patient cohorts when there was insufficient data provided. Studies that included the same patient cohort for more than one publication were only included once in the meta-analysis.

### Quality assessment

Quality assessment was independently performed by two researchers (JB and RG) following the Quality Assessment of Diagnostic Accuracy Studies (QUADAS) tool, a validated tool for the quality assessment of diagnostic accuracy studies [9,10]. This tool was modified to ensure that it was applicable to the included validation studies, and included 12 of the 14 questions from the original QUADAS tool. Item 4 (time period between administering the rule and the occurrence of a subsequent fall) and item 12 (availability of clinical data following administration of the STRATIFY rule) were excluded as they were not deemed relevant to the STRATIFY rule. If no consensus was reached, studies were evaluated by a third independent reviewer (TF).

### Statistical methods

We used Stata version 10.1 (StataCorp College Station, Texas, USA), particularly the metandi commands for all statistical analyses. We have used this methodology in previous studies of this nature [11,12]. We used a cut point of ≥2 points to identify individuals at high risk of falls. Therefore we constructed a 2x2 table using this cut point and extracted the number of true positives, false positives, true negatives and false negatives for the STRATIFY CPR from each original validation study. We applied the bivariate random effects model to estimate summary estimates of sensitivity and specificity and their corresponding 95% confidence intervals. This approach was applied as it preserved the two-dimensional nature of the original data and took into account both study size and heterogeneity beyond chance between studies [13]. Sensitivity referred to the proportion of fallers correctly classified as high fall risk. Specificity was the proportion of non-fallers correctly classified as low fall risk. It was not possible to calculate pooled estimates using the bivariate model with less than four studies.

Individual and summary estimates of sensitivity and specificity for the STRATIFY CPR were plotted in a receiver operating characteristic (ROC) graph, plotting the rules sensitivity (true positive) on the y axis against 1-specificity (false negative) on the x axis. We also plotted the 95% confidence region and 95% prediction region around the pooled estimates to illustrate the precision with which the pooled values were estimated (confidence ellipse around the mean value) and to illustrate the amount of between study variation (prediction ellipse).

We evaluated heterogeneity visually using the summary ROC plots and statistically by using the variance of logit transformed sensitivity and specificity, with smaller values indicating less heterogeneity among studies. We used Bayes theorem to estimate the post-test probability of a fall, by multiplying the pre-test odds by the likelihood ratio, where pre-test odds are calculated by dividing the pre-test probability by (1-pre-test probability) and the post-test probability equals post-test odds divided by (1 + post-test odds). We completed sensitivity analyses to explore the effect of methodological features (as determined by the QUADAS tool) on the diagnostic accuracy of the STRATIFY CPR.

## Results

### Study identification

**Figure 1:**
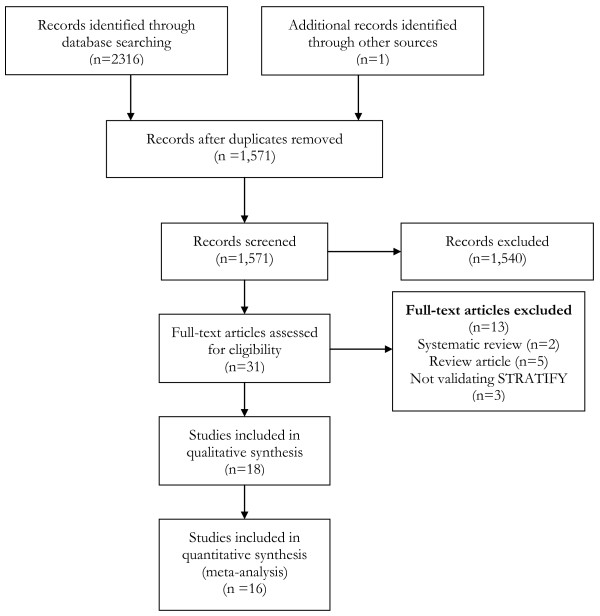
PRISMA flow diagram of search strategy.

A flow diagram of the search strategy is presented in Figure 1. Two researchers (JB, RG) screened all potential papers. The search strategy yielded 2,317 of articles, of which 2286 were excluded based on title or abstract. Eighteen of the remaining 31 articles met our inclusion criteria and were included in the systematic review [2,6,8,14-28]. For the purposes of the meta-analysis, we excluded two studies; the original study by Oliver as the unit of analysis in this paper was number of falls as opposed to number of falls per individual [8] and a study by Barker and colleagues because they used a cut point of ≥3 to identify patients at high risk of falling [14]. We included 16 different studies with 17 cohorts of patients in the meta-analysis because one paper contained data on two separate patient groups [18]. In a further study, we only included a subgroup of patients (aged ≥65 years) in our meta-analysis [6].

### Study characteristics

The characteristics of the studies are contained in Table 1. Six studies were based in the United Kingdom [8,14-18], five in Australia [2,19-22], two in Canada [23,24], one in Germany [25], one in Belgium [6],one in the Netherlands [26], one in France [27] and one in Italy [28]. The size of patient cohort in the included studies ranged from 44 [22] to 5,489 [21] participants. In total, 11,378 patients were included in the meta-analysis. We used the proportion of fallers (prevalence 6.27%, range 1.1%-41.3%) as a measure of baseline risk and heterogeneity in included studies and settings.

**Table 1:** Characteristics of studies included in the review

**Study**	**Participants: n, sex, mean age (range)**	**Time frame patients followed up**	**Population type**	**Person who administered tool**	**Person who recorded the fall**	**Number of falls**	Number of individuals who fell*
**Oliver et al. 1997**[8] United Kingdom (Local validation)	n = 217 79.5 years	8 weeks	Geriatric unit, rehabilitation unit	Nurse	NR - recorded in ward incident book	71	Unreported
**Oliver et al. 1997**[8] United Kingdom (Remote validation)	n = 331 83 years	8 weeks	Geriatric unit, rehabilitation unit	Nurse	NR - recorded in ward incident book	79	Unreported
**Walsh et al. 2011**[2] Australia	n = 130 51 men; 79 women 75 years (29-97)	Until discharge	Medical/surgical inpatients	Research team	Principal investigator	14	7 (5.4%)
**Marschollek et al. 2011**[25] Germany	n = 46 81.3 years	One year	Geriatric inpatients	Interdisciplinary geriatric team	Interdisciplinary Geriatric care team	Unreported	19 (41.3%)
**Barker et al. 2010**[14] United Kingdom	n = 263 137 men; 126 women 61.32 years	Until discharge	Medical/surgical inpatients	Research nurse	Nurse	44	23 (8.7%)*
**Webster et al. 2010**[20] Australia	n = 788 77.7 years	Until discharge	Hospital inpatients ≥65 years	Research team	Staff member recorded into Incident Report Database	Unreported	72 (9.1%)
**Vassallo et al 2008**[16] United Kingdom	n = 200 77 men; 123 women 80.9 years	3 weeks	Rehabilitation unit	Clinician	Nurse	Unreported	51 (25.5%)
**Milisen et al. 2007**[6] Belgium	n = 1602† 627 men; 975 women 79.3 years	Until discharge	Hospital inpatients ≥65 years	Bedside nurses	Nurse	1968	123 (7.7%)
**Kim et al. 2007**[21] Australia	n = 5489 2842 men; 2647 women 55 years	Until first fall, discharge or death	Hospital inpatients ≥ 18 years	Research nurse	Nurse	Unreported	60 (1.1%)
**Wijnia et al. 2006**[26] The Netherlands	n = 120 75 men; 45 women 74.5 years	13 weeks	Nursing home	Nurse	Nurse	Unreported	36 (30%)
**Smith et al. 2005**[17] United Kingdom (Inpatient study)	n = 225	28 days	Stroke rehabilitation units	Nurse	Staff member using incident report form	Unreported	53 (23.6%)
**Haines et al. 2006**[19] Australia (Phase 1)	n = 122 38 men; 84 women 79 years	Until discharge	Hospital inpatients	Project researcher	Staff member (using incident report form)	59	26 (21.3%)
**Vassallo et al. 2005**[15] United Kingdom	n = 135 49 men; 86 women 83.8 years (56-100)	Until discharge	Hospital inpatients	Clinician	Nurse	29	22 (18%)
**Jester et al. 2005**[18] United Kingdom	n = 90 20 men; 70 women (60-81 years)	Unreported	Hospital inpatients	Unreported	NR – information was obtained from medical/nursing/therapy notes,Incident report forms	Unreported	5 (5.6%)
**Hill et al. 2004**[22] Australia	n = 44 26 men; 18 women 79.8 years (64-101)	Unreported	Geriatric rehabilitation unit	Clinician	Ward staff	Unreported	7 (15.9%)
**Papaioannou et al. 2004**[24] Canada	n = 620 282 men; 338 women 78 years	Unreported	Hospital inpatients ≥65 years	Nurse	Nurse	77	34 (5.5%)
**Coker et al. 2003**[23] Canada	n = 432	Until discharge	Geriatric rehabilitation unit	Nurse	Nurse	Unreported	111 (25.7%)
**Chiari et al. 2002**[28] Italy	n = 1,181 507 men; 604 women (65-104 years)	2 months	Hospital inpatients	Project researcher	Staff member	Unreported	51 (4.3%)
**Bailleux, 2006**[27] France	n = 155 55 men; 100 women 80.3 years	Until discharge	Geriatric rehabilitation unit	Project researcher	Staff member (using incident report form)	Unreported	36 (23.2%)

*Mean weighted prior probability = 6.27%, NR - not reported.

**Cut point of ≥3 used on the STRATIFY rule.

†Indicates a subset of the patient cohort was used in our analysis.

### Study quality

**Figure 2:**
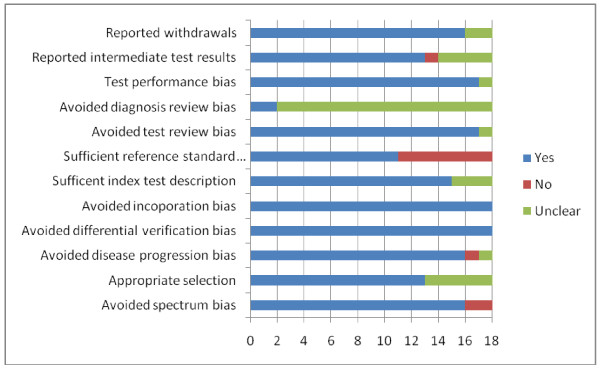
Quality assessment of included articles.

The summary diagram of the quality assessment is shown in Figure 2. The overall quality of the included studies was moderate to good, with only two of the included articles [17,18] not avoiding spectrum bias. However, seven of the eighteen included studies did not give sufficient description of the reference standard, in this case, the definition of a fall [15,16,18,21,22,25,28]. In addition, it was unclear whether diagnosis review bias was avoided, as sixteen studies did not explicitly state whether the occurrence of a fall was interpreted without knowledge of the results of the STRATIFY rule [2,6,14,16-28]. Furthermore, two studies did not clearly report details of withdrawals from the patient cohort [20,23].

### Diagnostic test accuracy of all included studies

The pooled sensitivity, specificity and the respective variance of the logit transformed sensitivity and specificity for the seventeen studies included in the meta-analysis are displayed in Table 2. These findings indicate that the STRATIFY rule has limited diagnostic accuracy at a cut point ≥2. However, the CPR is more useful at ruling out rather than ruling in falls in individuals classified as low risk, with a higher pooled sensitivity (0.67, 95% CI 0.52-0.80) than specificity (0.57, 95% CI 0.45-0.69).

**Table 2:** Summary estimates of sensitivity, specificity, and positive and negative likelihood ratios for all included studies and for sensitivity analyses at a cut point of ≥2

**Application of STRATIFY rule**	**No. of studies (patients)**	**Sensitivity (95% CI)**	**Variance Logit Sensitivity (95% CI)**	**Specificity (95% CI)**	**Variance Logit Specificity (95% CI)**
**All studies**	17 (n = 11,378)	0.67 (0.52-0.80)	1.49 (0.63-3.53)	0.57 (0.45-0.69)	1.04 (0.49-2.21)
**Studies with spectrum bias excluded**	14 (n = 11,063)	0.66 (0.54-0.76)	0.69 (0.28-1.72)	0.61 (0.51-0.69)	0.49 (0.22-1.09)
**Studies with no definition ‘fall’ excluded**	10 (n = 4,193)	0.61 (0.42-0.78)	1.26 (0.43-3.68)	0.65 (0.55-0.74)	0.42 (0.15-1.16)
**Studies with a high prevalence of falls (>10%)**	9 (n = 1479)	0.58 (0.41-0.73)	0.94 (0.32-2.77)	0.58 (0.43-0 .71)	0.76 (0.28-2.08)
**Studies with a low prevalence of falls (<10%)**	8 (n = 9899)	0.75 (0.42-0.93)	1.12 (-0.31-2.55)	0.63 (0.43-0.79)	0.53 (-0.28-1.33)

**Figure 3:**
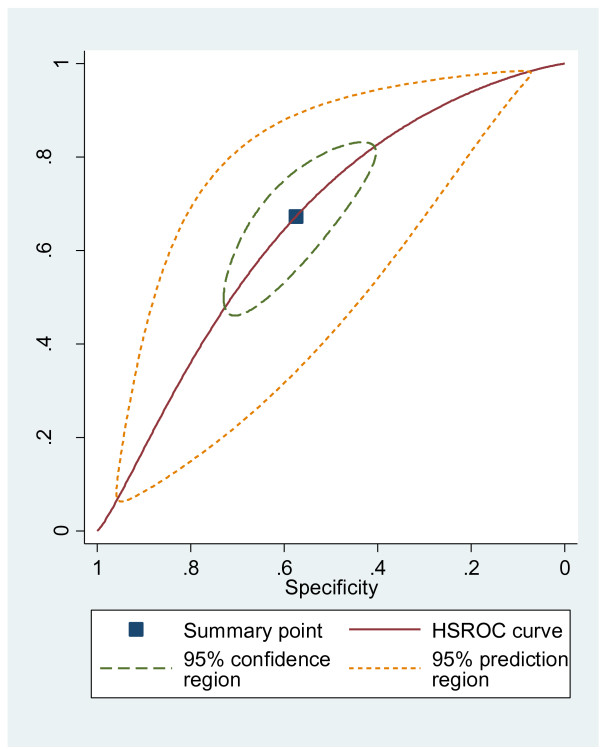
Receiver operating characteristic graph with 95% confidence region and 95% prediction region for all included studies (n=17) at a cut point ≥2.

Individual and summary estimates of sensitivity and specificity for all of the studies included in our meta-analysis, the 95% confidence region and 95% prediction region are presented in the summary ROC graph (Figure 3). The 95% confidence region was broad, reducing the precision of studies in the pooled estimate. The 95% prediction region (amount of variation between studies) was also wide suggesting heterogeneity between studies.

### Sensitivity analysis

**Figure 4:**
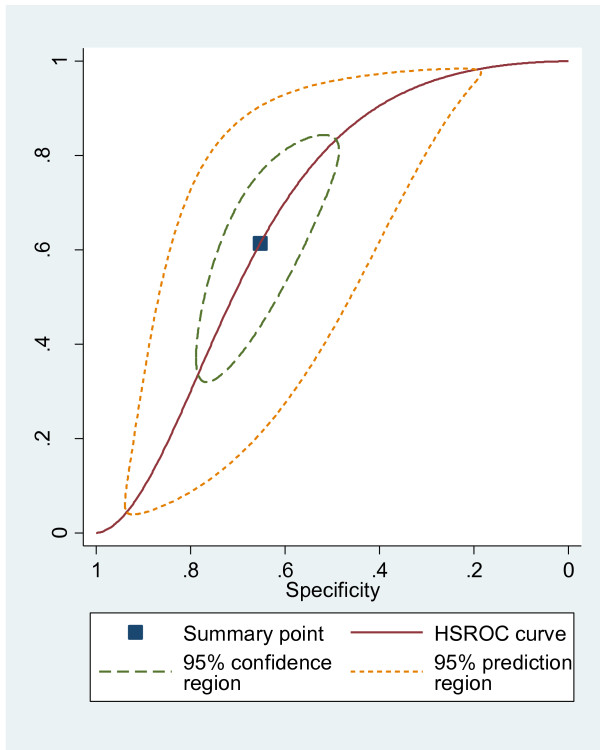
Receiver operating characteristic graph with 95% confidence region and 95% prediction region for studies that provide a definition of a fall (n=9) at a cut point ≥2.

We completed a sensitivity analysis, excluding two articles (comprising three patient cohorts) with evidence of spectrum bias [17,18]. The summary estimates of sensitivity (0.66, 95%CI 0.54-0.76) and specificity (0.61, 95%CI 0.51-0.69) were unchanged. We also completed a sensitivity analysis excluding seven articles (comprising eight patient cohorts) studies where an explicit definition of falls was not provided [15-18,22,25,28], with broadly similar results (Table 2 and Figure 4). Finally, we examined the clinical value of the rule in low and high prevalence settings (<10% vs >10% respectively). The STRATIFY rule performed better in a low prevalence setting with a pooled sensitivity of 0.75 (95%CI 0.42-0.93) and a pooled specificity of 0.63 (0.43-0.79). However, the 95% confidence interval was large, indicating that there was less precision for the pooled estimates in this setting.

### Bayesian analysis

Using Bayes’ theorem, the post-test probability of a fall across the different settings and subgroups are presented in Table 3. Most notable, a score of ≥ 2 points on the STRATIFY rule doubled the pre-test probability of a subsequent fall in a low prevalence setting. A STRATIFY score of ≥2 increased the pre-test probability of a subsequent fall from 6.3% to almost 10% and a score of <2 reduced the probability of a subsequent fall to 3.7% across all clinical settings. The positive likelihood ratio of 1.58 (95% CI 1.34-1.86) indicated that the STRATIFY CPR was not optimal for identifying individuals at high risk of falls across a variety of clinical settings.

**Table 3:** Post-test probability of a fall in patients classified as high risk (≥2 points) and low risk (<2 points) using the STRATIFY score

**Application of STRATIFY rule**	**Pre test probability (%)**	**+ LR (95% CI)**	**Post test probability (%) + LR**	**-LR (95% CI)**	**Post test probability (%) -LR**
**All studies**	6.27% (5.84%-6.74%)	1.58 (1.34-1.86)	9.58% (8.26%-11.09%)	0.57 (0.43-0.75)	3.67% (2.8%-5.03%)
**Studies with spectrum bias excluded**	5.93% (5.51%-6.39%)	1.67 (1.43-1.95)	9.53% (8.29%-10.93%)	0.56 (0.45-0.71)	3.44% (2.75%-4.29%)
**Studies with no definition ‘fall’ excluded**	11.9% (10.96%-12.92%)	1.76 (1.48-2.12)	19.27% (16.62%-22.24%)	0.59 (0.41-0.85)	7.4% 5.25%-10.32%
**Studies with a high prevalence of falls (>10%)**	24.41% (22.24%-26.68%)	1.39 (1.21-1.60)	30.98% (28.08%-34.03%)	0.72 (0.59-0.87)	18.82% (15.99%-22.03%)
**Studies with a low prevalence of falls (<10%)**	3.57% (3.22%-3.95%)	2.03 (1.69-2.44)	6.99% (5.89%-8.28%)	0.39 (0.18-0.86)	1.42% (0.65%-3.08%)

## Discussion

### Statement of principal findings

This systematic review demonstrates that the diagnostic accuracy of the STRATIFY rule is limited at the widely used cut point of ≥2 and should not be used in isolation for identifying individuals at high risk of falls in clinical practice. The sensitivity analysis which examined the performance of the rule in different settings and subgroups also showed broadly comparable results, indicating that the STRATIFY rule performed in a similar manner across a variety of different ‘at risk’ patient groups in different clinical settings.

### Context of previous studies

Our findings are in keeping with that of a previous systematic review that pooled the results of four studies. The results of the previous systematic review demonstrated that the diagnostic accuracy of the STRATIFY CPR was limited with overall sensitivity and specificity estimates of 0.67 (95% CI 0.61–0.74) and 0.51 (95% CI 0.43–0.59) respectively. This systematic review that pools data from 17 different studies adds to the existing body of evidence and further quantifies the rules’ lack of clinical value across a range of different settings [5].

The original derivation paper by Oliver et al. had some limitations [7]. Firstly, the nature of the study design used to derive the STRATIFY rule was not optimal and subject to bias in terms of choosing appropriate controls and determining exposure. In addition, the unit of analysis in the original paper was the number of episodes of falls that occurred during the study period. In essence, each fall was regarded as a new incident and patients who fell several times were included as multiple data entries. Using the cut point of ≥2 points, the sensitivity of the STRATIFY rule was reported to be >0.90 in the patients included in the two narrow validation cohorts. However, all of the subsequent validation studies reported the number of individuals who fall as the unit of analysis, thus eliminating the clustering effect of more than one fall in an individual patient. This may have contributed to the significantly lower estimates of sensitivity and specificity in these studies. In addition, the weighting of the predictor variables in the STRATIFY rule require further evaluation. The original study assigned a simple unweighted scoring system to the STRATIFY rule. Papaioannou et al. [24] modified the weighting of the STRATIFY items in a Canadian setting demonstrated the modified rule had a sensitivity of 91.2% and specificity of 60.2% at a cut point of ≥9 points. However, the modified rule has not been validated in independent studies.

Our systematic review examined the clinical value of the STRATIFY score at traditional cut point of ≥2. However, the accuracy of the rule may be improved by using a different cut point to identify ‘at risk’ patients; therefore future research should examine the clinical value of the rule at different cut points. The predictor variables included in the STRATIFY CPR also need to be reconsidered in future research. A systematic review by Ganz examined the predictive value of risk factors for subsequent falls and reported that variables included in the STRATIFY rule such as visual impairment, decreased activities of daily living, and agitation did not consistently predict falls across studies [3].

### Strengths and weaknesses

This study pooled data from a broad range of studies and settings, enhancing the generalisability of its findings. We examined the quality of the studies using a validated method for assessing the quality of such studies. In addition, sensitivity analyses examined the effect of important clinical and methodological variables. The results of this study should be interpreted in the context of the study limitations. We considered the methodological quality of the included studies to be reasonable, however it was unclear whether sixteen of the studies avoided diagnosis review bias, by not explicitly stating if the occurrence of a fall was interpreted without knowledge of a STRATIFY score [2,6,14,16-28]. Furthermore, seven of the included articles did not provide a definition of what was considered to be a ‘fall’, and this may have impacted on the STRATIFY CPRs performance. However, our findings showed little difference in the pooled estimates when restricting analysis, thus supporting the overall results.

### Clinical implications

Falls risk screening tools are a common element in many hospital-based programmes. These tools are used to identify patients at high risk for falls and to facilitate the effective delivery of appropriate interventions to such patients [19]. Inaccuracy of falls screening tools has lead to inappropriate distribution of resources, contributing to varying degrees of success and failure of falls prevention strategies. It is essential to establish the diagnostic accuracy of such tools and identify alternative tools that may be able to identify patients at risk of falling more accurately. A recent clinical review paper examined different falls assessment tools in older people and suggests that the STRATIFY and the modified STRATIFY rule should be used in isolation to assess falls risk in the hospital and home environment [29]. Our systematic review does not support this statement as the totality of evidence demonstrates that the diagnostic accuracy of the STRATIFY rule is limited and it should not be used in its current format as a screening instrument to guide falls prevention interventions. In terms of the clinical utility of the STRATIFY CPR, this systematic review showed that it is more useful to rule out falls in patients who score <2 (low risk individuals). Therefore, we suggest that the STRATIFY rule should not be used in isolation, but rather could be used in Step 1 of a falls management strategy, to assist clinicians in identifying which patients require a more thorough multifactorial falls assessment. Step 2 should comprise the multifactorial assessment items with weighted diagnostic importance: gait and balance, cognition, medication use, basic and instrumental activities of daily living, visual acuity and home environment. These multifactorial assessments can serve to inform Step 3 of the falls management process and guide the allocation of particular interventions such as physiotherapy, occupational therapy and interventions to target inappropriate medication use.

Research has also focused on the diagnostic accuracy of alternative screening tools to assess falls risk including the timed Up and Go Test and QuickScreen tools. However, the totality of evidence on relation to their predictive accuracy warrants further investigation. The accuracy of the clinical judgment of nurses has also been examined and has been reported to be comparable to some current falls screening tools [19]. Screening for falls risk using clinical judgment has been achieved in a number of ways including rating patients as being at high, medium, or low risk of falls or asking staff whether the patient would benefit from a specific falls prevention intervention [19]. The predictive value of patients self judgment has also been suggested as a method of screening [3]. Further investigation is warranted to determine the merits and reliability of these judgments, in patients and professional disciplines. In the meantime, clinicians should apply caution when screening for falls risk using these methods until more robust evidence is available.

## Conclusion

This systematic review has shown that the diagnostic accuracy of the STRATIFY CPR is limited and should not be used in isolation for identifying individuals at high risk of falls.

## Competing interests

The authors declare that they have no competing interests.

## Authors’ contributions

All authors were involved in the study conception and design. JB performed a systematic search of the literature. Both JB and RG screened potential articles, evaluated the methodological quality of studies, acquired data for analysis, performed statistical analysis and interpretation of data and drafted the paper. TF critically revised the draft manuscript. All authors read and approved the final manuscript.

## Funding sources

This work was supported by the Health Research Board (HRB) of Ireland through the HRB Centre for Primary Care Research under Grant HRC/2007/1.

## Pre-publication history

The pre-publication history for this paper can be accessed here:

http://www.biomedcentral.com/1471-2296/13/76/prepub

## Supplementary Material

Additional file 1 **Table S1.** STRATIFY falls risk assessment tool. Click here for file
