# Supplementary material for: Diagnostic accuracy of the STRATIFY clinical prediction rule for falls: A systematic review and meta-analysis
Source: BMC Fam Pract. 2012 Aug 7;13:76. doi: 10.1186/1471-2296-13-76 (PMC3460792; doi:10.1186/1471-2296-13-76)
Supplement: Additional file 1 — Table S1. STRATIFY falls risk assessment tool. [file 1471-2296-13-76-S1.doc]

**Table 1: STRATIFY falls risk assessment tool**

Choose one of the following options which best describes the resident’s level of capability when **transferring** from a bed to chair *(From Barthel Index)*

| **Answer** | **Score** |
| --- | --- |
| Unable | 0 |
| Needs major help | 1 |
| Needs minor help | 2 |
| Independent | 3 |

Choose one of the following options which best describes the resident’s level of **mobility**

| **Answer** | **Score** |
| --- | --- |
| Immobile | 0 |
| Independent with the aid of a wheelchair | 1 |
| Walks with the aid of one person | 2 |
| Independent | 3 |

**Total the transfer and mobility score and answer the next question**

*Note:* Yes = 1 (transfer and mobility score of 3 or 4).

No = 0 (transfer and mobility score of 0, 1, 2, 5 or 6)

1. Is the combined transfer and mobility score 3 or 4?

| **Answer** | **Score** |
| --- | --- |
| YES | 1 |
| NO | 0 |

2. Has the resident had any falls in the last 3 months?

| **Answer** | **Score** |
| --- | --- |
| YES | 1 |
| NO | 0 |

3. Is the resident visually impaired to the extent that everyday function is affected?

| **Answer** | **Score** |
| --- | --- |
| YES | 1 |
| NO | 0 |

| **Answer** | **Score** |
| --- | --- |
| YES | 1 |
| NO | 0 |

4. Is the resident agitated?

5. Do you think the resident is in need of especially frequent toileting?

| **Answer** | **Score** |
| --- | --- |
| YES | 1 |
| NO | 0 |

| **Total of questions 1 – 5** |  |
| --- | --- |
